# Marginal Micronutrient Intake in High-Performance Male Wheelchair Basketball Players: A Dietary Evaluation and the Effects of Nutritional Advice

**DOI:** 10.1371/journal.pone.0157931

**Published:** 2016-07-06

**Authors:** Lena Grams, Guadalupe Garrido, Jorge Villacieros, Amelia Ferro

**Affiliations:** 1 Department of Health and Human Performance, Faculty of Physical Activity and Sport Sciences, Technical University of Madrid, Madrid, Spain; 2 Department of Sports, Faculty of Physical Activity and Sport Sciences, Technical University of Madrid, Madrid, Spain; Universidad Europea de Madrid, SPAIN

## Abstract

Wheelchair basketball has evolved into a high-performance sport over several years, and small variations in player performance can determine the difference between winning and losing. Therefore, adequate micronutrient intake may influence this difference if performance-promoting macronutrient intake and physical fitness are equal between teams. Seventeen elite male wheelchair basketball players belonging to the Spanish National Team participated in this study. Macro- and micronutrient intake were determined using a food-weighing diary over three consecutive days during three training camps in two consecutive years. Current Dietary Reference Intake levels were used to determine the adequacy of intake of seventeen micronutrients of particular interest for athletes. After categorizing the consumed foods into fourteen food groups according to the National Nutrient Database for Standard References (USDA) these groups were used to identify the best predictors of the adequacy of intake for each micronutrient. Total energy intake correlated positively with the adequacy of all micronutrient intake levels, except for vitamins A and E. Five B vitamins and phosphorus, selenium, and iron showed 100% adequacy. All other micronutrient intake levels were found to be inadequate, e.g., vitamin E (51% adequacy) and calcium (73%). The fruit, fish and cereal food groups were found to be predictors of adequate intake of most micronutrients. Together with energy intake (*p* = .009, η^2^ = 0.49), the intake of the fruit (*p* = .032, η^2^ = 0.39) and egg (*p* = .036, Kendall’s W = 0.42) food groups increased significantly over time, along with improved iodine (*p* = .008, *W* = 0.61) and magnesium (*p* = .030, *W* = 0.44) adequacy levels. Because the adequacy of micronutrient intake correlates positively with energy intake (R = 0.64, *p* < .001), a varied diet that includes cereals, fish and fruits is especially important for players with low levels of energy intake. Supplements may be a possible solution if adequate micronutrient intake cannot be achieved through regular dietary intake alone. However, dietary analyses should be conducted on a regular basis throughout the year to improve the nutritional knowledge of the athletes and assure adequate micronutrient intake.

## Introduction

In high-performance sports, differences in the factors that determine winning and losing can be small [1]. Hence, athletes should consider all factors that can help enhance their performance. In wheelchair basketball, three main factors are involved: the wheelchair, the athlete and the interactions between them [2]. As athletes undergo training programs, which are comparable to those of their able-bodied peers [3], the principles of sports nutrition should be considered to support effective training and to improve performance levels during training and competition [4, 5]. In addition to control the macronutrients energy distribution and timing of energy intake throughout the day, ensuring an adequate intake of micronutrients may also have a positive impact on performance [6].

Micronutrients, including vitamins and minerals, are required in small amounts and interact with one another to regulate physiological functions [4, 5]. For athletes, the roles of micronutrients in energy metabolism, blood and bone health and antioxidant functioning are extremely important, and the B vitamin group (e.g., thiamin, riboflavin, niacin, vitamin B_6_, folic acid and vitamin B_12_) and minerals (e.g., iodine, iron, magnesium and zinc) facilitate the conversion of macronutrients into energy and are therefore essential to athletes because of their high levels of energy expenditure [4, 5]. In athletes, red blood cell production is often increased, requiring an increased intake of iron, a component of hemoglobin, and folic acid and vitamin B_12_, which are also needed for the synthesis of DNA and RNA [7]. Furthermore, adequate levels of calcium, phosphorus and vitamin D ensure the maintenance of sufficient bone mineral density, helping to prevent stress fractures [7–9]. Certain antioxidants (vitamin A, C and E) and minerals (selenium and zinc) protect cells and tissues against damage from free oxygen radicals, which increase in number as a result of exercise [7]. Although athletes may have slightly higher micronutrient requirements because exercise stresses many of the metabolic pathways in which micronutrients are involved, no special recommendations have been described for these requirements [4, 5]. To date, there is no evidence of altered micronutrient requirements for wheelchair athletes, with the exception of those related to vitamin D, as osteoporosis is associated with vitamin D deficiency, which is common in athletes with spinal cord injuries (SCI) [10–12]. Therefore, we used the existing United States and Canadian Dietary Reference Intake (DRI) values to determine adequate micronutrient intake levels [13, 14].

Both the American College of Sports Medicine and the International Society of Sports Nutrition recommend a well-balanced diet to meet vitamin and mineral requirements [4, 5]. Nevertheless, athletes with restricted energy intake are at an increased risk of inadequate nutrient intake [4, 5]. Until now, very few studies have examined the energy intake patterns of male wheelchair athletes, and all of these studies have reported low energy intake levels within this group relative to the intake levels of able-bodied athletes [15–17]. Only one study, on male Canadian wheelchair athletes, has shown inadequacies in the micronutrient intake [16], and because inadequate micronutrient intake can weaken one’s immune system, increasing the risks of illness, the intake of nutrient-dense foods, that can improve the adequacy of micronutrients, could improve the health of these athletes.

Therefore, the aims of this study were to (1) evaluate micronutrient intake by elite wheelchair basketball players based on the latest Food and Nutrition Board (FNB) guidelines, (2) analyze which food groups are crucial for the adequacy of micronutrient intake, and (3) examine whether nutritional advice provided one month and again one year after the initial recommendations can improve diet quality.

## Methods

### Participants and study design

**Fig 1:**
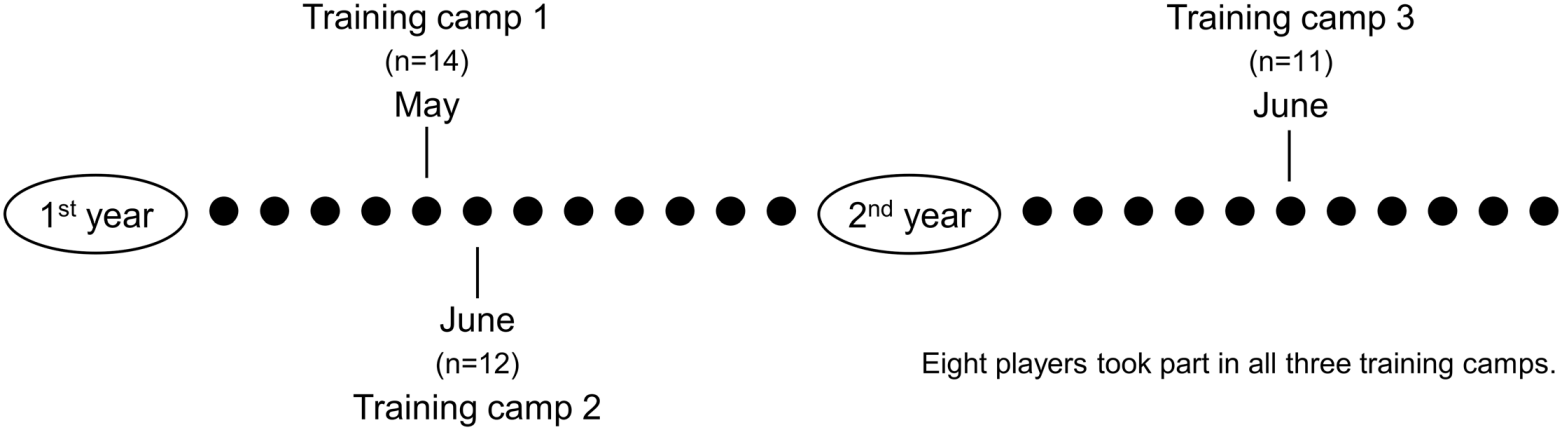
Timeline of all training camps (T1, T2 and T3).

Seventeen male wheelchair basketball players on the Spanish National Team participated in this study, which was conducted in Madrid (Spain) at the Higher Sport Council during three training camps held in the precompetitive season in two consecutive years. To compare the adequacy of micronutrient intake at the group level, data for all players participating in the camps in May (Training Camp 1 (T1), 14 players) and June of the first year (Training Camp 2 (T2), 12 players) and in June of the second year (Training Camp 3 (T3), 11 players) were used (Fig 1). Disabilities ranged from amputations to spina bifida and spinal cord injuries. The effectiveness of the nutritional advice given was determined in consultation with the eight players who participated in all the camps.

The Ethics Committee of the Technical University of Madrid (Spain) approved the study, which was conducted according to the Helsinki Declaration for research on humans [18]. Before participating, players provided their written consent.

### Anthropometrics

Height was measured using a stadiometer (DKSH Switzerland Ltd, Switzerland) to the nearest 0.1 cm in a standing position if possible, and for athletes who were unable to stand, the formula recommended by Canda [19] was applied to estimate height based on arm span and seated height. Body weight was determined using a calibrated scale (Kern, Twister Medical, Spain) to the nearest 0.1 kg with athletes wearing minimal clothing.

### Diet composition

Diet compositions were estimated using a food-weighing diary (1 g accuracy; Mettler-Toledo S.A.E, Barcelona, Spain) over three consecutive days during each camp. Experienced researchers weighed and recorded each food component at breakfast, lunch and dinner. The players orally reported the types and quantities of snacks consumed outside of the main meal times and the time of day they were consumed. Likewise, supplement intake for each player was determined, and players provided leaflets, labels or packaging information. To analyze micronutrient intake from food alone, the intake due to supplements was determined separately. During each camp, the players ate together at the Centre of Higher Sport Council, selecting their food from a buffet offering a wide variety of food options. Occasionally some players chose to eat at home or outside the hotel area and orally reported the types and quantities of food they have consumed. The software program DIAL version 2 (Alce Ingeniera, Madrid, Spain) was used to determine the nutrient composition of the meals. The recipes of each meal were discussed with the kitchen staff to identify the separate food components so that they could be assigned to food groups, and recipes not already included in the food composition database were added. The weights of the food components were then entered into DIAL to analyze diet composition and categorize the components into fourteen food groups (S1 Table).

### Adequacy of micronutrient intake

Ten vitamins (vitamin A, thiamin, riboflavin, niacin, vitamin B_6_, folic acid and vitamin B_12_ vitamin C, vitamin D and vitamin E) and seven minerals (calcium, iodine, iron, magnesium, phosphorus, selenium and zinc) of special relevance to athletes were examined [4, 5]. Individual intake was determined using the probability approach recommended by the American Dietetic Association [20, 21]. Furthermore, to determine intake adequacy at the group level, the Estimated Average Requirements (EAR)-cutpoint method with the latest DRI values from the FNB was applied [20–22]. According to the definition of the DRI, a probability of micronutrient intake of 50% corresponds with the EAR, and a probability of 100% corresponds with the Recommended Dietary Allowance (RDA) [21]. The Tolerable Upper Intake Level (UL) was set as the highest safe limit [14]. If the calculated probability of adequate intake for a micronutrient was stable at 100%, the amount in grams was used as the intake level instead of the probability. This was the case for niacin, riboflavin, vitamin B_6_, vitamin B_12_, iron, phosphorus and selenium. In our study, the sum of all micronutrients with a probability of at least 50% (adequacy_SUM_) was calculated as the overall adequacy score to determine longitudinal improvement levels.

### Nutritional advice

After each of the three nutritional evaluations (T1, T2, T3), individual written reports and personal interviews were conducted to optimize diet composition and micronutrient intake (i.e., daily amounts, food group variation, pre- and post-exercise snacks). All evaluations were held under the same conditions and during the same time of the year. Individual feedback was provided after each evaluation.

Resting energy expenditures were estimated according to Abel et al. [23], and energy expenditures were calculated using written activity protocols and values provided by Collins et al. [24] for athletes with SCI and by Abel et al. [23] and Bernardi et al. [25] for amputees.

### Statistics

Normal distributions were tested for using the Shapiro-Wilk test and the Kolmogorov-Smirnov test. Pearson and Spearman correlations were computed for parametric and non-parametric data, respectively, to determine the linear relation between energy and micronutrient intake. Stepwise multiple linear regressions were conducted for each micronutrient intake as a dependent variable, and food groups were used as independent variables so that we could determine the relationship between micronutrient intake and dietary variation. Differences in micronutrient intake between all three evaluations were tested via analyses of variance (ANOVA) with repeated measurements for parametric data or via Friedman tests for non-parametric data. For post hoc tests we used paired Student’s t-tests for parametric data and Wilcoxon tests for non-parametric data, using Bonferroni corrections with partial eta-squared (η^2^) and Kendall’s W to represent the effect size, respectively. Parametric data are given as the mean ± standard deviation values and non-parametric as median [minimum; maximum] values. Significance was accepted at *p* < .05, and all tests were performed using SPSS version 22 (IBM Corp., Armonk, NY, USA).

## Results

### Participants

Characteristics for each player (5 amputees and 12 athletes with spina bifida or SCI, age 30 [22;41] years) are shown in Table 1.

**Table 1:** Player characteristics and diet composition shown as the mean values over the number of diaries.

Player	Number of diaries	Height (cm)	Weight (kg)	BMI (kg/m^2^)	SCI or amputee
1	1	189	84.0	23.5	Amputee
2	3	181	102.8	31.4	Amputee
3	2	187	88.5	25.3	Amputee
4	3	186	76.8	22.2	Amputee
5	2	183	75.3	22.5	Amputee
6	3	182	88.2	26.6	SCI
7	2	178	76.8	24.2	SCI
8	3	170	73.9	25.6	SCI
9	2	171	61.9	21.2	SCI
10	1	190	82.3	22.8	SCI
11	3	175	75.3	24.6	SCI
12	3	184	69.9	20.6	SCI
13	3	170	46.7	16.1	SCI
14	1	186	74.1	21.4	SCI
15	1	176	69.0	22.3	SCI
16	1	173	65.0	21.7	SCI
17	3	183	70.6	21.1	SCI
		182 [170;190]	75.5±13.5	23.3±3.7	

BMI, body mass index; SCI, spinal cord injury

### Diet composition

Overall, 37 food diaries were examined. Mean energy intake was 2,673±485 kcal/d (1,597 to 3,651 kcal/d) and 36.8±10.5 kcal/kg (24.6 to 63.5 kcal/kg). Macronutrient intake relative to body mass was 3.9 [1.8;8.1] g/kg for carbohydrates and 1.7±0.6 g/kg for protein, excluding supplements. The percentage of energy derived from fat was 33.7±5.5%.

### Micronutrient intake

**Fig 2:**
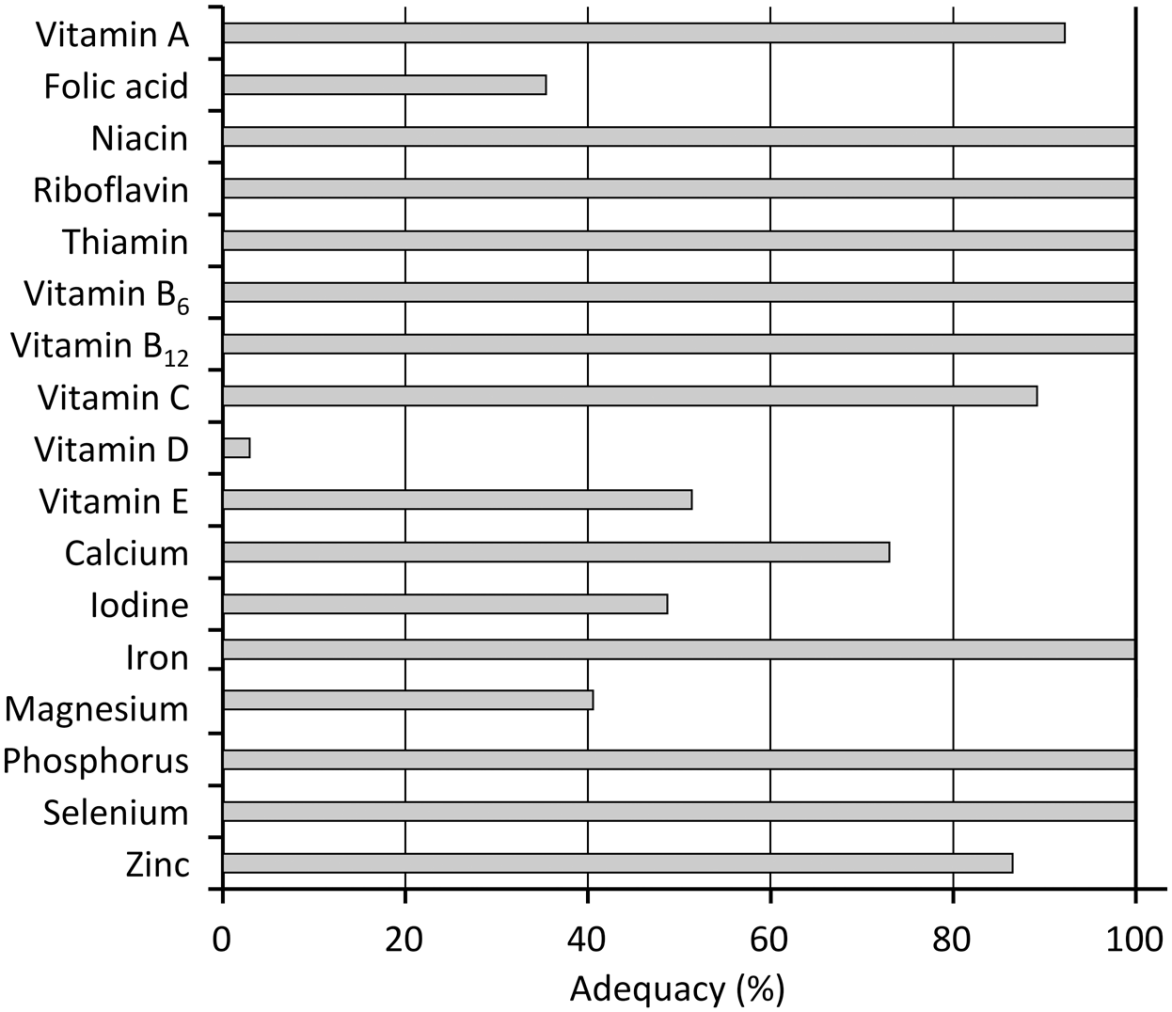
Probability of adequate micronutrient intake.

The estimated adequate intake probabilities at the group level are shown in Fig 2. The levels shown exceed the RDA for niacin, riboflavin, vitamin B_6_ and vitamin B_12_ and for various minerals (iron, phosphorus, and selenium) for all the players. No player exceeded the UL for any micronutrient. Adequacy_SUM_ was found to be 13.2±2.0, with no player achieving adequate intake levels for all 17 micronutrients but with 35% taking in adequate levels for 16 micronutrients.

One player used supplements on all three occasions, achieving adequate calcium and vitamin D levels for Training Camp T1 and adequate intake levels for all micronutrients and calcium for Training Camp T2. Two players used mineral supplements on one occasion, achieving levels of calcium above adequacy.

The correlation of total energy intake with adequacy_SUM_ and micronutrient intake adequacy is shown in Table 2.

**Table 2:** Correlation between total energy intake and the probability of adequate micronutrient intake.

	Correlation coefficient	*p*-Value
Adequacy_SUM_	0.64	<0.001
Vitamins		
Vitamin A	0.29	0.087
Folic acid	0.50	0.002
Niacin (mg)^a^	0.59	<0.001
Riboflavin (mg)^a^	0.63	<0.001
Thiamin	0.44	0.006
Vitamin B_6_ (mg)^a^	0.59	<0.001
Vitamin B_12_ (μg)^a^	0.53	<0.001
Vitamin C	0.33	0.044
Vitamin D	0.54	<0.001
Vitamin E	0.29	0.082
Minerals		
Calcium	0.44	0.006
Iodine	0.64	<0.001
Iron (mg)^a^	0.53	<0.001
Magnesium	0.55	<0.001
Phosphorus (mg)^a^	0.65	<0.001
Selenium (μg)^a^	0.41	0.013
Zinc	0.59	<0.001

^a^ The probability of adequate intake is constant at 100%

Players with higher adequacy_SUM_ levels consumed more fruit, dairy, fat, oil, and cereals (Table 3).

**Table 3:** Beta coefficients of stepwise multiple linear regressions with the probability of adequate micronutrient intake as a dependent variable and energy intake levels from different food groups as independent variables.

	R^2^	Cereals	Legumes	Vegetables	Fruits	Dairy	Meats	Fish	Eggs	Sugars and sweets	Fats and oils	Beverages	Prepared meals	Appetizers	Sauces
Adequacy_SUM_	0.72	0.27			0.58	0.22					0.42				
Vitamins															
Vitamin A	0.25							0.36					-0.30		
Folic acid	0.71			0.30	0.66				0.40	0.33					-0.25
Niacin (mg)^a^	0.87	0.24		0.29	0.21		0.66	0.38	0.21						
Riboflavin (mg)^a^	0.54	0.43							0.49						0.38
Thiamin	0.26	0.51													
Vitamin B_6_ (mg)^a^	0.49				0.35		0.30		0.44						
Vitamin B_12_ (μg)^a^	0.85					0.30	0.55	0.64	0.26				0.26		
Vitamin C	0.50				0.30								-0.38	-0.41	
Vitamin D	0.24							0.49							
Vitamin E	0.62				0.50						0.51				-0.27
Minerals															
Calcium	0.40					0.63									
Iodine	0.61				0.36	0.41					0.46			-0.27	
Iron (mg)^a^	0.55	0.39		0.35	0.48		0.40								
Magnesium	0.67	0.51			0.42			0.27							
Phosphorus (mg)^a^	0.75	0.38				0.34	0.29	0.41	0.30						
Selenium (μg)^a^	0.66	0.37				-0.21		0.60							
Zinc	0.21	0.46													

^a^ The probability of adequate intake was constant at 100%

### Effects of nutritional advice

Anthropometric data and diet compositions for the eight players who took part in all three evaluations are shown in Table 4. Although body weight and BMI levels changed significantly over time, post hoc tests showed no significance. Total energy intake increased significantly during the evaluation at T3 relative to values recorded for T1 (*p* = .017) and T2 (*p* = .046). Similar results were found for fruit (*p* = .032, *η*^*2*^ = 0.39) and egg intake levels (*p* = .036, Kendall’s *W* = 0.42), although significant post hoc test results were not found.

**Table 4:** Anthropometric data and diet compositions for the eight players who took part in all three evaluations.

Parameter	Training Camp T1	Training Camp T2	Training Camp T3	*p*-Value	η^2^
Age (years)	29.9±6.5	30.0±6.5	31.0±6.5	---	
Height (cm)	178.9±6.3		178.9±6.3	---	
Body weight (kg)	75.0±16.2	74.3±15.6	77.3±16.5	0.029	0.40
BMI (kg/m^2^)	23.4±4.7	23.1±4.5	24.1±4.7	0.032	0.39
Total energy intake (kcal/d)	2441±341^b^	2446±588^a^	2968±453^a^^,^^b^	0.009	0.49
Carbohydrate (g/kg)	3.1 [2.8;7.1]	3.5 [1.8;8.1]	4.9 [2.7;7.4]	0.197	
Protein (g/kg)	1.6±0.7	1.5±0.5	1.9±0.7	0.071	
Fat (%)	35.1±4.7	33.7±4.8	32.1±5.9	0.599	
Cholesterol (mg)	401 [263;947]	317 [226;518]	669 [216;992]	0.012	0.25

^a^ T2 vs. T3 *p* < .05,

^b^ T1 vs. T3 *p* < .05 BMI, body mass index

**Fig 3:**
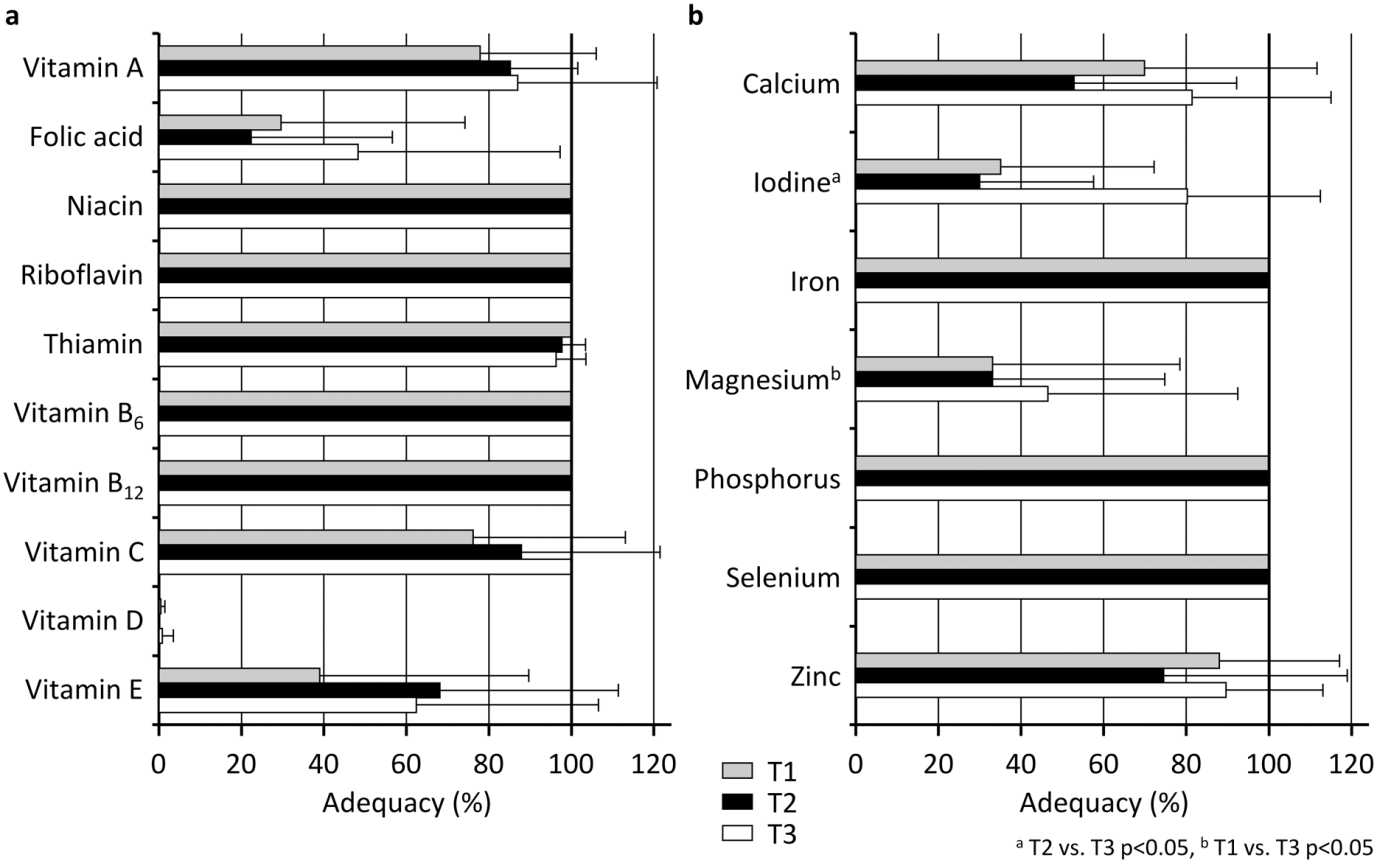
Probabilities of adequate vitamin (a) and mineral intake (b) for the eight players who took part in all three evaluations (T1, T2 and T3).

The probabilities of adequate micronutrient intake for the three evaluations are shown in Fig 3. Iodine (*p* = .008, *W* = 0.61; T1-T2 n.s., T2-T3 *p* = .035, T1-T3 *p* = .045) and magnesium (*p* = .030, *W* = 0.44; T1-T2 n.s., T2-T3 *p* = .788, T1-T3 *p* = .023) showed significant differences, with three players over the EAR for iodine at T1, one over at T2 and seven over at T3 and with three over the EAR for magnesium at T1, two over at T2 and three over the RDA at T3.

The adequacy_SUM_ values were 12.5±2.4 at T1, 12.3±1.8 at T2 and 14.0±1.7 at T3 (*p* = .039, *η*^*2*^ = 0.37, T1-T3 *p* = .043).

## Discussion

Our study reveals a relationship between total energy intake and adequate micronutrient intake in male wheelchair basketball players. Additionally, it shows that a variety of food groups are needed for one to achieve adequate micronutrient intake.

Although a higher total energy intake among able-bodied athletes should result in adequate micronutrient intake [4, 5], it is less certain whether sufficient energy intake can help Paralympic athletes to achieve this goal. Athletes with SCI in particular often have a lower muscle mass than able-bodied athletes as a result of paralysis [26], which is caused by a reduction in sympathetic nervous system activity below the level of the lesion [27]. Together with lower activity levels or even immobility in the affected limbs, this leads to less active muscle mass, and therefore, their energy expenditure is likely to be lower than that of their able-bodied peers [28, 29]. Likewise, their total energy intake may be equally reduced to maintain energy balance, possibly resulting in inadequate micronutrient intake. Krempien and Barr [17] reported on the energy intake levels of male Canadian wheelchair athletes, and Goosey-Tolfrey and Crosland [15] reported similar results for male British wheelchair athletes. Both groups were found to have lower intake levels than able-bodied professional basketball players [30]. Although the mean total energy intake levels found in the present study are higher than those found for comparable disabled athletes [15, 17], the values ranged from 1,597 to 3,651 kcal/d, highlighting the need for individualized nutritional advice for athletes with different disabilities and even among those who play the same sport. Similarly, the participants in the present study varied in terms of body weight, explaining the similarly high variability in energy intake relative to body weight, which ranged from 25 to 64 kcal/kg.

However, total energy intake is not the only factor that affects the adequacy of micronutrient intake. For example, similar energy intake levels of 2,558 kcal/d and 2,479 kcal/d were found in our study between two players and resulted in the adequate intake of ten micronutrients for one player and sixteen for the other, with only vitamin D showing inadequate intake levels, which will be discussed later. Consequently, a varied diet is also important [4, 5]. In our study, intake of eleven of the fourteen food groups we examined (see detailed information in S1 Table) showed a positive correlation with adequate intake levels for at least one micronutrient. From these groups, four (cereals, fruits, dairy, and fats and oil) accounted for 72% of the variance in the overall adequacy of micronutrient intake (Table 3). Fruits were found to be associated with five vitamins and three minerals, fish were associated with four vitamins and three minerals, and cereals were associated with three vitamins and five minerals. Dairy was the only significant source of calcium intake found, even though we expected additional sources (e.g., specific vegetables or legumes) to play an important role as the food available in the buffet included all the necessary food groups to support optimal nutritional requirements.

Compared with the levels found in the study by Krempien and Barr [16] on male Canadian athletes with SCI, micronutrient intake levels were generally found to be higher in our study. This observation can partly be explained by the higher energy intake levels used (2,673±486 kcal/d) relative to those employed by Krempien and Barr (2,285 kcal/d) [16]. However, Krempien and Barr [16] did not consider issues relating to dietary variety, which we found to have a considerable effect. Comparisons between energy intake levels of non-athlete adults with SCI revealed even greater differences, with Perret and Stoffel-Kurt [31] reporting an energy intake of 1,775 kcal/d and lower absolute micronutrient intake levels, with the exception of those for calcium.

The dietary intake levels of our participants showed a high adequacy for 13.3±2.1 of the 17 micronutrients examined. Micronutrients involved in bone health, such as calcium, magnesium and vitamin D, showed low adequacy levels, and the levels of phosphorus were high in relation to those of calcium [32], which could negatively affect progress among wheelchair athletes who are susceptible to osteoporosis [11]. Only one player, an amputee, used a multivitamin and mineral supplement that included calcium and vitamin D, which provided adequate levels of both micronutrients for this individual.

However, the DRI for vitamin D represents intake levels under minimal sun exposure conditions, and in the summer, the endogenous synthesis of vitamin D in Madrid, Spain, (40° in latitude) is achieved with 10 minutes of daily sunlight exposure. Nonetheless, limited sun exposure can occur because of the season and at latitudes higher than 35°. In such cases, calcium and vitamin D supplements should be considered during fall and winter and especially for athletes presenting low total energy intake levels.

In addition to improve performance, micronutrients play an important role in enhancing immune function, and illness can result in players being absent from training and competition [15, 33]. To prevent the spread of illness and to support immune system health, ensuring adequate levels of vitamins A, D, E, C, B_6_, and B_12_, as well as folic acid, iron and zinc should be of great concern [34, 35]. In our study, vitamin B_6_ and vitamin B_12_ intake levels were found to be adequate as animal food groups were well represented and no player followed a vegetarian diet. Vitamin A, vitamin C, iron and zinc intake levels were above their EAR. Various food groups served as significant sources of vitamin A and iron, although only fruits served as sources of vitamin C, and only cereals served as sources of zinc. Regarding the low adequacy levels of vitamin E and folic acid intake, a higher intake of nutrient-dense legumes may prove beneficial, as legumes are good sources of these micronutrients. However, due to the low legume intake in this study, legumes were not found to serve as a significant source of any micronutrient. Although digestion is slower in players with SCI [36] and certain food groups induce flatulence, it seems likely that some legumes or vegetables (e.g., boiled greens, potatoes, carrots and alliums) offered as purees would be easier to digest and could be beneficial nutrient-dense foods. In this regard, individual solutions should be found [6] and discussed between the athlete and nutritionist.

Nutritional advice was found to increase the overall adequacy of micronutrient intake after one year, which can be attributed to a higher total energy intake and to a more varied diet characterized by the higher fruit and egg intake of our participants. Considering the marginal differences found after the second training camp (T2), which was only four weeks after the first (T1), it appears that eating habits take time to change and that advice should be given more frequently than it was in our study to achieve adequate micronutrient intake. Even a nutrition education program involving three sessions over four months did not improve micronutrient intake among able-bodied professional handball players [37], despite the fact that the motivation of elite athletes should be high to support effective training and to improve performance levels during training and competition. Despite lower levels of cholesterol intake found after four weeks (T2), our data revealed an increasing trend after one year (T3) (Table 4). This finding could partly be explained by a higher egg intake, which on the one hand led to a high protein intake but on the other hand led to a cholesterol intake that was above the recommended amount (Table 4). Our recommendations to increase the consumption of fat-free and fat-reduced dairy products were not followed over a full year. Consequently, nutritional advice seems more effective when provided over a shorter timeframe.

### Study Limitations

Although the DRI values were used to study the intake adequacy of all micronutrients, our analysis conducted over three consecutive days may have been too short to fully analyze the requirements for some micronutrients [21]. Other potential reasons for intake deficiencies (e.g., the bioavailability of micronutrients) were not examined in this study and should be considered before vitamin or mineral supplements are recommended. Blood analyses must also be conducted when confirming inadequate intake. In regards to vitamin D status, varying levels of sun exposure across seasons have a considerable effect on the endogenous synthesis, and therefore, supplements may prove beneficial during fall and winter but are likely unnecessary during spring and summer. Finally, the micronutrient contents in food are unstable, so the actual levels taken in may be different than we estimated, and some of our data on nutrient content may have been inaccurate as food databases do not include the chemical compositions of all available foods.

## Conclusion

The adequacy of the micronutrient intake by elite male Spanish wheelchair basketball players is positively related to their total energy intake. However, a variety of food groups must be included in one’s diet to achieve adequate levels for the most important micronutrients, and nutrient-dense food groups such as fruit are associated with more micronutrients than less nutrient-dense groups. As some micronutrients showed inadequate intake levels, additional nutritional advice should be provided to avoid adverse effects resulting from personal dislike of certain food groups, and the consumption of beneficial foods (especially vegetables and legumes prepared in ways that are easy to digest) should be promoted as some of these groups, legumes in particular, were only consumed in minimal amounts. In addition, shorter time frames between nutritional advice are more successful than longer periods, such as one year.

Food variety with special attention to nutrient-dense foods is especially important in players with low energy intake. Consequently, it might be necessary to use supplements for players with SCI, especially for micronutrients involved in bone health. Nutritional monitoring in Paralympic athletes should be incorporated into the regular routine of their training periods throughout the year so that a short time frame between repeated sessions of receiving nutritional advice can be used to raise their knowledge of the benefits of sports nutrition.

## Supporting Information

S1 Table Food groups and their components. (DOCX)
