# Supplementary material for: Marginal Micronutrient Intake in High-Performance Male Wheelchair Basketball Players: A Dietary Evaluation and the Effects of Nutritional Advice
Source: PLoS One. 2016 Jul 6;11(7):e0157931. doi: 10.1371/journal.pone.0157931 (PMC4934691; doi:10.1371/journal.pone.0157931)
Supplement: S1 Table — (DOCX) [file pone.0157931.s001.docx]

**S1 Table. Food groups and their components.**

| **Food group** | **Component** |
| --- | --- |
| Cereals | Grains and flour  Breakfast cereals  Bread  Pasta  Low fat cookies  Low fat pastries |
| Legumes | Dry legumes  Canned legumes  Derivatives of legumes |
| Vegetables | Fresh vegetables  Frozen vegetables  Canned vegetables  Natural vegetables juices  Canned vegetables juices  Tuber, root vegetables  Canned tuber, root vegetables  Mushrooms  Canned mushrooms |
| Fruits | Fresh fruits  Derivatives of fruits  Dried fruits  Nuts  Natural fruit juices |
| Dairy | Milk  Yoghurt and fermented milk  Cheese  Milk desserts  Milk shakes  Cream |
| Meats | Beef  Pig  Lamb  Poultry  Guts  Sausages  Smoked meat  Other meat |
| Fish | White fish  Blue fish  Frozen fish  Canned fish  Smoked fish  Seafood and derivatives  Canned seafood and derivatives  Salted fish |
| Eggs | Eggs |
| Sugars and sweets | Sugar  Chocolate  Sweets  Full fat pastries  Other sweets  High fat cookies |
| Fats and oils | Vegetable Oils  Butter and margarines  Other fats |
| Beverages | Soft-drinks  Coffee and tea  Herbal tea  Isotonic beverages  Commercial juices  Other soft-drinks without alcohol  Alcoholic drinks |
| Prepared meals | Prepared commercial dishes ready to eat  (e.g. Pizza, Fast food) |
| Appetizers | Appetizers  (e.g. Potato chips, Tortilla chips) |
| Sauces | Commercial and handmade sauces  Spices |
